# An effective and well-tolerated strategy in recurrent and/or metastatic head and neck cancer: successive lines of active chemotherapeutic agents

**DOI:** 10.1186/1471-2407-14-504

**Published:** 2014-07-10

**Authors:** Julien Péron, Valentine Polivka, Sylvie Chabaud, Marc Poupart, Philippe Ceruse, Antoine Ramade, Didier Girodet, Philippe Zrounba, Jérôme Fayette

**Affiliations:** 1 Department of Medical Oncology, Centre Léon Bérard, University of Lyon, Lyon, France; 2 Hospices Civils de Lyon, Service de Biostatistique, F-69003 Lyon, France; 3 CNRS UMR 5558, Laboratoire de Biométrie et Biologie Evolutive, Equipe Biostatistique-Santé, Villeurbanne, France; 4 Department of Biostatistics, University of Lyon, Centre Léon Bérard, Lyon, France; 5 University of Lyon, Department of Otorhinolaryngology, Hôpital de la Croix-Rousse, Lyon, France; 6 Department of Otorhinolaryngology, Centre Hospitalier Lyon Sud, University of Lyon, Pierre-Bénite, Lyon, France; 7 Department of Otorhinolaryngology, Hôpital E Herriot, University of Lyon, Lyon, France; 8 Department of Otorhinolaryngology, Centre Léon Bérard, University of Lyon, Lyon, France

**Keywords:** Recurrent and/or metastatic head and neck squamous cell carcinoma, Chemotherapy, Survival analysis, Treatment outcome, Drug administration schedule

## Abstract

**Background:**

The combination platinum, 5-fluorouracil (5-FU) and cetuximab is the standard first-line regimen of recurrent and/or metastatic head and neck squamous cell carcinoma (HNSCC). Due to the toxicity of this treatment, alternative therapies are often offered to patients. The aim of this study was to evaluate the overall survival obtained with a first line chemotherapy adapted to patients functional status and the administration of all active drugs within successive lines of chemotherapy.

**Methods:**

This series included a total of 194 patients with recurrent and/or metastatic HNSCC treated from 2006 to 2011 in a single institution where the administration of successive lines of chemotherapies has been the standard clinical approach. Treatment was administered according to clinical practice guidelines.

**Results:**

Most patients received at least two treatment lines. Only 11 patients (6%) were treated with a combination of cisplatin, 5-FU and cetuximab in front line, but most patients received at least one platinum-based regimen (n = 154 patients, 78%); 162 (82%) received taxanes, 36 (18%) received 5-FU, 27 (14%) received capecitabine, 67 (34%) received methotrexate and 134 (68%) received cetuximab. The median overall survival was 9.8 months (95% CI: 8.1-11.4 months) and reached 13.1 months among the subgroup of 131 patients eligible for inclusion in a clinical trial.

**Conclusion:**

The survival outcomes of patients treated in the first-line setting with chemotherapy regimens adapted to their functional status, followed by several subsequent regimens were comparable with published outcomes of patients treated by platinum, 5-FU and cetuximab.

## Background

Despite progress in the primary treatment of head and neck squamous cell carcinoma (HNSCC) by combining chemotherapy with surgery, radiation therapy and supportive care, recurrence rates range from 30 to 50%. For the treatment of recurrent and/or metastatic HNSCC, platinum-based combination chemotherapy has been the standard first-line treatment, providing a median overall survival (OS) of six to nine months [1-5]. However, since the combination of cisplatin, 5-fluorouracil (5-FU) and cetuximab was shown to be superior to that of cisplatin, 5-FU and placebo in the EXTREME phase III trial [5], it became the new standard for first-line treatment of recurrent and/or metastatic patients. Response rates significantly increased from 20 to 36%, together with progression-free survival (PFS) (from 3.3 to 5.6 months) and OS (from 7.4 to 10.1 months; HR = 0.80; 95% CI: 0.64-0.99; p = 0.04). Only 6% of patients treated with chemotherapy alone received cetuximab after the study was completed. Thus, cetuximab has become a key drug for the treatment of recurrent and/or metastatic HNSCC; however, its most effective place in the treatment strategy remains to be determined.

Only patients in good general condition (Karnofsky scale ≥ 70%) and with adequate organ function were included in the EXTREME trial. Nevertheless there were more cases of febrile neutropenia (9 patients vs. 1, p = 0.02), cutaneous toxicity (9% of grade 3–4) and allergic reaction in the group of patients treated with cetuximab. Given the efficacy shown by cetuximab administered as a single agent [6] or in combination with less toxic chemotherapeutic agents, such as taxanes [7,8], first-line treatment with the highly toxic combination of cetuximab and platinum-based chemotherapy might be avoided without loss of efficacy. After failure of first-line platinum-based chemotherapy, further chemotherapy regimens are now available. Indeed, taxanes alone [9,10] or taxanes in combination with cetuximab [7,8], capecitabine [11,12] or methotrexate [1] are available as subsequent treatment lines.

Given the low tolerability of the combination cisplatin-5FU plus cetuximab, most patients in our institution did not receive cetuximab as first-line treatment, but later, during follow-up. Therefore, we retrospectively analyzed the outcomes of patients treated in a single institution, where the administration of successive lines of chemotherapies has been the standard clinical approach.

## Methods

### Data retrieval and file selection

We retrospectively reviewed the data of all patients with histologically confirmed recurrent and/or metastatic HNSCC and treated by chemotherapy at a single institution between March 2006 and August 2011. Previous chemotherapy could have been administered for the treatment of the primary tumor (induction chemotherapy or in combination with radiation therapy). Written consent was obtained from each patient according to the institutional practice and french regulation. This study was approved by the ethics committee of the Centre Léon Bérard cancer center. Patient data were collected in accordance with CNIL rules (the French authority for protection of patient data) and kept anonymous.

### Treatment

**Figure 1:**
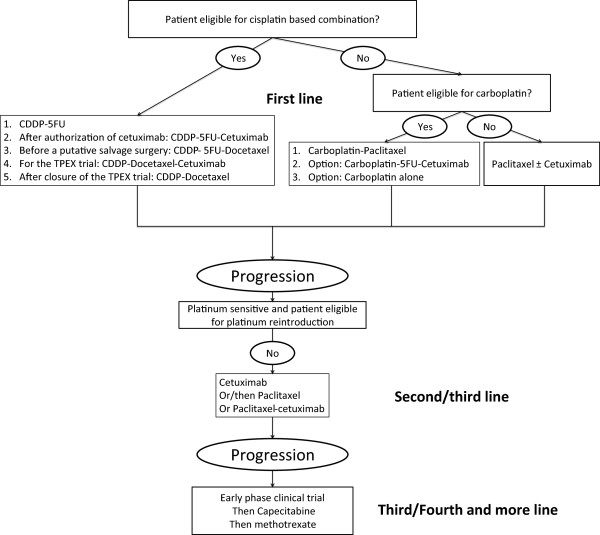
Chemotherapy guidelines for patients with recurrent and/or metastatic head and neck squamous cell carcinoma.

Treatment was administered according to clinical practice guidelines (Figure 1). The choice of first-line therapy depended on the date of treatment onset, the patients’ condition and the efficacy or residual toxicity of previous platinum-based chemotherapy when used in treatment for localized disease. Recent local clinical practice guidelines recommended treating patients with platinum-sensitive disease (progressive disease more than 6 months after the end of the multimodal treatment including platinum for locoregional disease) with cisplatin in combination with taxanes alone, 5-FU alone, or 5-FU and cetuximab in the first-line setting. Common cisplatin ineligibility criteria were renal dysfunction (creatinine clearance of < 50 to 60 mL/min), poor performance status, advanced age (>70 to 75 years), and comorbidities (eg, severe neuropathy, congestive heart failure, hearing loss). Cisplatin-ineligible patients received a 3-weekly AUC5 carboplatin-based chemotherapy (Figure 1). Patients with highly impaired performans status (Karnofsky score below 50) or with highly impaired nutritional status could be ineligible to carboplatin. Carboplatin-ineligible patients and patients treated in the second-line setting who developed a resistance to platinum (progressive disease less than 6 months after the beginning of platinum) received a combination of paclitaxel ± cetuximab. If the first-line efficacy of the platinum-based chemotherapy was good, a combination of cisplatin and cetuximab could be proposed after progression. Subsequent lines consisted of inclusion in a clinical trial, methotrexate, or capecitabine, depending on previously given therapies and eligibility in a clinical trial. Each chemotherapy line was continued until disease clinical or radiological progression or significant toxicity. Chemotherapy regimens commonly used in our institution are described in the Table 1.

**Table 1:** Description of the common chemotherapy regimens used in the series

**Platinum based regimens (name and dose)**	**Platinum free regimens (name and dose)**
**Cisplatin** (100 mg/m^2^/3 weeks) + **5-FU** (1000 mg/m^2^/d, 5 days/3 weeks)	**Paclitaxel** (60 to 80 mg/m^2^/week) + **Cetuximab** (400 mg/m^2^ then 250 mg/m^2^/week)
**Cisplatin** (75 mg/m^2^/3 weeks) + **Docetaxel** (75 mg/m^2^/3 weeks)	**Paclitaxel** (80 mg/m^2^/week)
**Cisplatin** (75 mg/m^2^/3 weeks) + **Docetaxel** (75 mg/m^2^/3 weeks) + **Cetuximab** (400 mg/m^2^ then 250 mg/m^2^/week)	**Methotrexate** (40 mg/m^2^/week)
**Cisplatin** (75 mg/m^2^/3 weeks) + **Docetaxel** (75 mg/m^2^/3 weeks) + **5-FU** (750 mg/m^2^/d, 5 days/3 weeks)	**Capecitabine** (1,000 mg/m^2^ BID for 14 days/21 days)
**Cisplatin** (100 mg/m^2^/3 weeks) + **5-FU** (1000 mg/m^2^/d, 4 days/3 weeks) + **Cetuximab** (400 mg/m^2^ then 250 mg/m^2^/week)	
**Carboplatin** (AUC5/3 weeks) + **5-FU** (1000 mg/m^2^/d, 4 days/3 weeks) + **Cetuximab** (400 mg/m^2^ then 250 mg/m^2^/week)	
**Carboplatin** (AUC5/3 weeks) + **Paclitaxel** (80 mg/m^2^/week)	
**Carboplatin** (AUC5/3 weeks)	

5-FU = 5-Fluorouracil.

AUC = Area Under the Curve.

### Analysis

OS was defined as the time elapsed from the first dose of chemotherapy administered for the recurrent and/or metastatic disease. If death did not occur before the cut-off date, the patient was censored at the date of last valid assessment. The primary endpoint of the study was the estimation of the median OS. Inclusion of at least 150 patients was hoped to provide a sufficient precision in the estimation of the median OS (less than 6 months confidence interval range). Survival distributions were estimated by the Kaplan-Meier method. To evaluate the impact on OS of factors known to be relevant in recurrent and/or metastatic HNSCC prognosis, potential prognostic factors were included in univariate and multivariate Cox proportional hazard regression models.

Patients were considered ineligible for inclusion in a clinical trial at the time of the first line of chemotherapy for recurrent or metastatic disease if they had a performans status ≥ 2, life-threatening severe comorbidity, concomitant malignancy or disease progression within six months of curative-intent treatment for localized disease. Analyses were conducted among the entire study population and then restricted to patients virtually defined as eligible for inclusion in a clinical trial. All analyses were performed with SPSS v19.0 (SPSS Inc., Chicago, Illinois) and R (http://www.R-project.org/).

## Results

### Patient characteristics

Between March 2006 and August 2011, a total of 198 patients with histologically confirmed HNSCC received chemotherapy at the Centre Léon Bérard (Lyon, France) for a recurrent and/or metastatic disease. The characteristics of the study population are summarized in Table 2. The patients were mostly men (n = 171; 86%) with a median age of 61 years (ranging from 29 to 87 years) at the time of initiation of palliative chemotherapy.

**Table 2:** Baseline Demography of the Patient Population

**Median age, years [range]**	**61 [29–87]**
	***No. of patients *** **(%)**
**Sex**	
Female	27 (14)
Male	171 (86)
**Primary tumor site**	
Oral cavity	54 (27)
Oropharynx	55 (28)
Hypopharynx	47 (24)
Larynx	21 (11)
Other*	21 (11)
**Tumor stage at initial diagnosis**	
I	11 (6)
II	21 (11)
III	23 (12)
IVa	106 (54)
IVb	16 (8)
IVc	13 (7)
Unknown	8 (4)
**Initial treatment**	
Neoadjuvant platinum based chemotherapy	52 (26)
With Fluorouracil	43 (22)
With Taxanes	41 (21)
Surgery	142 (72)
Radiotherapy	168 (85)
Alone	73 (37)
With platinum	74 (37)
With cetuximab	20 (10)
With taxanes	1 (1)
**Pattern of disease**	
Primary metastatic	13 (7)
Primary locally advanced (palliative treatment)	8 (4)
Relapse	177 (89)
Local only	126 (63)
Metastatic +/- local	51 (26)
**Eligibility for inclusion in a clinical trial**	
Yes	131 (66)
No	67 (34)
PS ≥ 2	53 (27)
Relapse after initial therapy ≤ 6 months	27 (14)
Other concomitant malignancy	3 (2)
Major comorbidity	3 (2)
**PS at onset of palliative chemotherapy**	
0	28 (14)
1	117 (59)
≥ 2	53 (27)

*Four nasopharynx, 12 sinus tumor, 5 primitive adenopathies.

PS = Perormans Status.

The three most common sites of primary tumor were the oral cavity (n = 54; 27%), the oropharynx (n = 55; 28%) and the hypopharynx (n = 47; 24%). The tumors were advanced at the time of initial diagnosis, as 13 (7%) patients had metastases, 122 (62%) patients had stage IVa or IVb tumors, and 23 (12%) patients had stage III tumors.

Among patients treated in relapse (n = 177; 89%), initial treatment consisted of neoadjuvant platinum-based chemotherapy for 52 patients (26% of the whole cohort); 142 (72%) patients underwent surgery, and 168 (85%) received radiation therapy alone (n = 73; 37%) or in combination with chemotherapy (n = 75; 38%) or cetuximab (n = 20; 10%). Overall, 67 patients (34%) were ineligible for inclusion in a clinical trial, mainly because of a performance status ≥ 2 (n = 53; 27%), severe comorbidity or concomitant malignancy (n = 6; 3%) or disease progression within six months of curative-intent treatment for localized disease (n = 27; 14%).

### Mode of chemotherapy delivery

Data about chemotherapy delivery are summarized in Table 3. The most frequent first-line regimens chosen by physicians were those combining taxanes and carboplatin (n = 69; 35%) or cisplatin (n = 46; 23%), and mainly the combination of carboplatin and paclitaxel. Thirteen patients received the cisplatin, docetaxel and cetuximab combination evaluated in the TPEx trial [13]. Platinum without taxane combinations were administrated to only 28 patients (14%), including 11 patients (6%) treated with a combination of cisplatin, 5-FU and cetuximab, at the same schedule as in the EXTREME trial. Overall, 154 patients (78%) received at least one platinum-based regimen for the treatment of the recurrent and/or metastatic disease; 162 (82%) received taxanes, 36 (18%) received 5-FU, 27 (14%) received capecitabine, 67 (34%) received methotrexate, 134 (68%) received cetuximab, and 27 (14%) patients were included in at least one clinical trial for a novel agent (Table 3). On average, the patients eligible for inclusion in a clinical trial at the onset of palliative chemotherapy received a slightly higher number of chemotherapy lines and were exposed for a longer time to the different chemotherapeutic drugs (Table 3).

**Table 3:** Treatment Settings

**Number of chemotherapy lines in recurrent or metastatic setting**	**Among the whole cohort (N = 198)**	**Among patients eligible for inclusion in a clinical trial (N = 131)**
	**No. of patients (%)**	**No. of patients (%)**
1	74 (37)	33 (25)
2	47 (24)	37 (28)
3	44 (22)	33 (25)
4	23 (12)	19 (15)
5 or more	10 (5)	9 (7)
**Chemotherapy drug used**		
Taxanes	177 (89)	122 (93)
Including recurrent or metastatic setting	162 (82)	116 (89)
CDDP or Carboplatin	185 (93)	127 (97)
Including recurrent or metastatic setting	154 (78)	114 (87)
5FU	78 (39)	62 (47)
Including recurrent or metastatic setting	36 (18)	32 (24)
Cetuximab	144 (73)	103 (79)
Including recurrent or metastatic setting	134 (68)	100 (76)
Capecitabine in recurrent or metastatic setting	27 (14)	23 (18)
Methotrexate in recurrent or metastatic setting	67 (34)	39 (30)
Novel agent tested in a clinical trial in recurrent or metastatic setting	27 (14)	27 (21)
**Drug combination used in first line of the recurrent/metastatic disease**		
Platinum based combination	143 (72)	103 (79)
Cisplatin + taxanes	46 (23)	40 (31)
Carboplatin + taxanes	69 (35)	40 (31)
Platinum without taxanes	28 (14)	23 (18)
Including CDDP 5FU and cetuximab combination	11 (6)	10 (8)
Taxanes and cetuximab combination	14 (7)	8 (6)
Monotherapy	34 (17)	13 (10)
Clinical trials testing not approved regimens	7 (4)	7 (5)

CDDP = Cisplatin ; 5FU = 5-Fluorouracil.

### Efficacy

**Figure 2:**
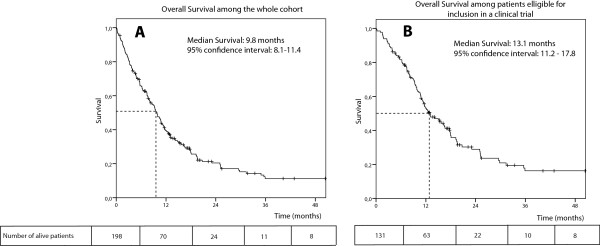
Kaplan-Meier estimates of overall survival among the whole cohort (A) and among patients eligible for inclusion in a clinical trial (B).

After a median follow-up of 33.4 months, a total of 156 patients (79%) had died. The median OS was 9.8 months (95% CI: 8.1-11.4 months) (Table 4). The outcomes of patients treated with first-line platinum-based chemotherapy were consistently acceptable. The median OS of patients treated with a combination of cisplatin and taxanes, carboplatin and taxanes, or a platinum-based combination without taxane were 14.2, 10.5 and 11.2 months, respectively (Table 5). Among the subgroup of patients eligible for inclusion in a clinical trial (n = 131; 66%), the median OS was 13.1 months (95% CI: 11.2-17.8 months) (Figure 2, Table 4), reaching up to 16.6 months (95% CI: 11.5-25.2 months) for the 68 patients treated with first-line cisplatin. A performans status ≥ 2, an age ≥ 60 years and a prior treatment with anti- epidermal growth-factor receptor were predictors of a poor OS in univariate analysis. Only a performans status ≥ 2 was independently associated with OS in multivariate analysis.

**Table 4:** Survival of patients

	**Median overall survival**
	**Time in months (95% CI)**
Among the whole cohort (N = 198)	9.8 (8.1-11.4)
Among patients eligible for inclusion in a clinical trial (N = 131)	13.1 (11.2-17.8)

**Table 5:** Survival of patients according to first line chemotherapy

	**Median overall survival**
	**Time in months (95% CI)**
Cisplatin + taxanes (N = 46)	14.2 (10.8-19.6)
Carboplatin + taxanes (N = 69)	10.5 (7.6-13.1)
Platinum without taxanes (N = 28)	11.2 (8.6-25.3)
Other chemotherapy (N = 48)	5.6 (4.3-8.1)
Clinical trials testing not approved regimens (N = 7)	5.7 (1.7 – NR)

NR = Non Reached.

### Safety

Five patients (3%) died as a result of adverse events at least possibly related to chemotherapy. Three of this patient died of febrile neutropenia after first line carboplatin and paclitaxel combination. All had severe comborbidities or impaired functional statues at the onset of chemotherapy (liver transplantation; anorexia leading an important weight loss; age > 80 years and impaired functional status). One patient died of an anaphylactic shock after the first course of paclitaxel and cetuximab. One patient died of an aspiration pneumonia on feeding tube a few days after the onset of cisplatin, 5-FU and docetaxel combination. The relation between the chemotherapy and the pneumonia is uncertain.

## Discussion

The results of this study challenge the use of the platinum, 5-FU and cetuximab combination for the first-line treatment of recurrent and/or metastatic HNSCC. Indeed, we showed here that the sequential use of all efficient chemotherapy regimens led to an OS of 9.8 months among a non-sorted patient population. In addition, the OS reached 13.1 months among patients without classical major exclusion criteria for clinical trials.

Today, the first-line standard treatment for recurrent and/or metastatic HNSCC is the combination of platinum, 5-FU and cetuximab, which is continued as maintenance therapy since the EXTREME trial demonstrated an overall response rate of 36%, a median PFS of 5.6 months, and a median OS of 10.1 months vs. 20%, 3.3 and 7.4 months without cetuximab, respectively. However, there were more severe adverse events in the arm of patients treated with cetuximab. The EXTREME trial only included patients in good general condition (with a Karnofsky > =70%), with adequate organ function, and with a relapse-free interval of at least six months since the last chemotherapy for the treatment of the local disease [5]. Because of the high toxicity, many patients with impaired nutritional or functional status are not eligible for this regimen, and other therapeutic strategies should be offered to these patients.

In our series, platinum-based combination was the favored first line when patients were fit enough, to avoid subsequent platinum-ineligibility related to disease progression. Patient ineligible for cisplatin and for the platinum, 5-FU and cetuximab combination were treated with a carboplatin-based front line therapy. The combination of carboplatin and weekly paclitaxel showed response rates of 48 to 53% and median survival rates of 8 to 12.8 months in Phase II trials [14,15]. In addition, the tolerability was better than with a traditional three-weekly administration [14,16]. This combination could then be proposed to patients with an impaired general condition (with a performance status of 2 or >2). For these reasons, carboplatin and weekly paclitaxel was the standard carboplatin-based regimen in this series. For patients ineligible or resistant to a platinum therapy, less toxic first line regimens have been offered to patients. The efficacy of taxanes as monotherapy [9,10] or in combination with cetuximab [7,8,17] after failure of platinum-based chemotherapy has been shown in several studies, with a response rate of 30% as monotherapy and a response rate of 38 to 54% for the combination. The tolerability profiles were good for both monotherapy and the combination. Thus, platinum, taxanes and cetuximab appear to be drugs of choice for the treatment of recurrent and/or metastatic HNSCC, and could be used either in combination or as successive therapies.

Since the approval of cetuximab for the treatment of recurrent and/or metastatic HNSCC, the comparison between several lines of single agent vs. combination chemotherapy regimens has never been performed in a clinical trial. In 1992, in a trial comparing cisplatin and 5FU as single agents and in combination, the response rate to the combination was higher but survival did not improve. In other cancer subtype, it appears more important for patients to receive all the active agents at some point during the course of treatment rather than the order or combination in which the drugs are received [18-20]. Considering the high rate of comorbidities, impaired nutritional status, and impaired functional status of patients with recurrent and/or metastatic HNSCC, they should be treated with less toxic strategies. Thus, successive chemotherapy lines could be an effective treatment option. The higher response rate observed with combination strategy [5] has been shown to improve the functional status of patients [21]. Moreover some patients in this series never received cetuximab. It appears that some patients would never receive any second line treatment given a rapid disease progression after failure of the first line regimen. The successive chemotherapy lines strategy might then be more suited for patients with low cancer-related symptoms, and with low tumor burden.

In this series, only 6% of the patients were treated in first line with the combination of platinum, 5-FU and cetuximab. First line therapy was adapted to patients’ general status and comorbidities. A major objective pursued by treating oncologists was to give all the active agents to a maximum of patients, explaining the high rate of patients receiving platinum, taxanes, cetuximab, methotrexate and 5-FU at least once. The estimation of patient’s outcomes in this series might be biased, given the retrospective nature of the study, the high heterogeneity of the patients and of the treatments.

Supportive care performed during and after the chemotherapy could have increased patients outcomes, as early integration of supportive care has been associated with survival benefit in another cancer setting (lung cancer) [22], but teoretically could be transposed to head and neck cancer and deserve further studies. However because all consecutive patients treated in our institution have been included in this series, it might be a relevant estimation of “real life” patient outcomes. Even if a formal comparison is not possible between our retrospective study and the results of clinical trials, the OS in our study was higher than those obtained with a combination of platinum, 5-FU and cetuximab in first line [23]. The high number of successive chemotherapy line (39% of patients received 3 lines or more) might explain patients good outcomes. Because of the heterogeneity of the treatment described in this study, and because of the limitations of a retrospective study, we did not assess the adverse events rates of the successive chemotherapy lines strategy. However, the tolerability of multiple successive chemotherapy regimens should compare favorably with multiple-drugs combinations [5]. In this series, deaths related to chemotherapy toxicities were uncommon (3%) despite the high number of chemotherapy lines administered to patients. The two first lines were involved in all of these deaths.

The short PFS observed with single-agent regimens [9-11,23,24] in first line does not necessarily indicate a failure of the global treatment strategy, if the progression can be identified before the occurrence of severe cancer-related symptoms, and if a switch to another chemotherapy is possible at progression. Therefore, a treatment strategy, including several lines of chemotherapy, should be formally compared with first-line combinations of multiple drugs in randomized controlled trials. Overall survival and a symptom scale should be the two major endpoints in such trials [21], since progression-free survival should systematically favored the combination arm. With the increased number of innovative and active agents, strategy trials should become more and more important. The administration of successive chemotherapy lines could spare patients from severe toxic effects, but might not be as efficient as combination strategy in controlling symptoms for patients with severe cancer-related symptoms or with rapidly progressive disease. The combination of cisplatin-5FU plus cetuximab might then be preferred in first-line given its higher response rate. The eligibility criteria for a treatment strategy with successive chemotherapy lines were not investigated in this retrospective series, but only patients without severe cancer-related symptoms, with low tumor burden and who are eligible for a close follow-up were treated with this strategy. Given the increasing number of efficient drugs for the treatment of recurrent and/or metastatic HNSCC, the design of future clinical trials in this setting should define the successive lines to be used after failure of the first line therapy (when the investigational drug is evaluated in first line). The primary endpoint might then be the time to strategy failure, defined as the sum of PFS of the two or three planned treatment course. In conclusion, our results are very encouraging, but subsequent efficient drugs are now strongly needed.

## Conclusions

The survival outcomes of patients treated in the first-line setting with chemotherapy regimens adapted to their functional status, followed by several subsequent regimens were comparable with published outcomes of patients treated by platinum, 5-FU and cetuximab. The administration of successive chemotherapy lines could spare patients from severe toxic effects, but might not be as efficient as combination strategy in controlling symptoms for patients with severe cancer-related symptoms or with rapidly progressive disease. It is a reasonable treatment option for patients with impaired functional status or severe organ failure to avoid the high toxicity of combination strategy.

## Competing interest

The authors declare that they have no competing interest.

## Authors’ contributions

JP and JF participated in the design of the study. JP, VP and SC performed the statistical analysis. JP and JF participated in the coordination and helped to draft the manuscript. MP, PC, AR, DG, PZ and JF included patients in the study. All authors read and approved the final manuscript.

## Pre-publication history

The pre-publication history for this paper can be accessed here:

http://www.biomedcentral.com/1471-2407/14/504/prepub
